# After-hour trauma-radiograph interpretation in the emergency centre of a District Hospital

**DOI:** 10.1016/j.afjem.2022.04.001

**Published:** 2022-06-06

**Authors:** Yi-Ying Melissa Liu, Suzanne O'Hagan, Frederik Carl Holdt, Sa'ad Lahri, Richard Denys Pitcher

**Affiliations:** a Division of Radiodiagnosis, Department of Medical Imaging and Clinical Oncology, Faculty of Medicine and Health Sciences, Stellenbosch University, Cape Town, South Africa; b Division of Emergency Medicine, Department of Family and Emergency Medicine, Faculty of Medicine and Health Sciences, Stellenbosch University, Cape Town, South Africa

**Keywords:** Plain films, X-rays, Trauma imaging, Fracture detection, Missed fracture

## Abstract

**Introduction:**

Plain radiographs remain a first-line trauma investigation. Most trauma radiographs worldwide are reported by junior doctors. This study assesses the accuracy of after-hour acute trauma radiograph reporting by emergency centre (EC) doctors in an African district hospital.

**Methods:**

An institutional review board approved retrospective descriptive study over two consecutive weekends in February 2020. The radiologist report on the admission radiographs of adult trauma patients was compared with the initial EC interpretation. The accuracy, sensitivity, specificity, positive predictive value (PPV) and negative predictive value (NPV) for EC interpretation were calculated with 95% confidence intervals (95%CI). The association between reporting accuracy and anatomical region, mechanism of injury, time of investigation, and the number of abnormalities per radiograph was assessed.

**Results:**

140 radiographs were included, of which 49 (35%) were abnormal. EC doctors recorded (95%CI) 77% (69-84%) accuracy, 38% (25-54%) sensitivity, 97% (91-99%) specificity, 86% (65-95%) PPV and 76% (71-80%) NPV. Performance was associated with the anatomical region (p=0.02), mechanism of injury (p=<0.01) time of day (p=0.04) and the number of abnormalities on the film (p=<0.01). The highest sensitivity was achieved in reports of the appendicular skeleton (42%) and in the setting of simple blunt trauma (62%). Overall accuracy was in line with the range (44%-99%) reported in the international literature.

**Discussion:**

Accurate reporting of acute trauma radiographs is challenging. Key factors impact performance. Further training of junior doctors in this area of clinical practice is recommended. Future work should focus on assessing the impact of such training on reporting performance.

## African relevance

• There is a global shortage of radiologists, which is most pronounced in resource-limited environments.
• Non-radiologist reporting of trauma radiographs is common in resource-limited settings, including most African countries.
• Trauma is the second leading killer of people in Africa. Non-radiologists report most trauma radiographs in Africa. Emergency Centre (EC) doctors in Africa require high proficiency in trauma radiograph interpretation.
• This study provides the most comprehensive analysis to date of trauma-radiograph reporting by non-radiologists in Africa.
• No such study has been performed in Africa previously; this is important for assessing the adequacy of basic radiology training in the emergency medicine program.

## Introduction

Due to their affordability, availability, and relatively low ionizing radiation exposure, plain radiographs remain a crucial first-line trauma investigation, particularly in resource-limited settings. For the past fifty years, the global radiological workload has increased at a faster rate than the supply of radiologists, compounded by the introduction of new imaging techniques, such as computed tomography, magnetic resonance imaging and fluoroscopically guided interventions. There has thus been an increasing global shortage of radiologists, with most trauma radiographs worldwide being reported by junior, inexperienced doctors [1], [2], [3], [4].

Trauma care creates a “perfect storm” for medical errors, with high workload, unstable patients, incomplete histories, time-critical decisions, multi-tasking, interdisciplinary involvement, and junior staff working outside normal hours [5]. It is therefore inevitable that errors will be made and that some diagnoses missed [1]. Failure to detect fractures is the most common error in Accident and Emergency Units [6]. Approximately 10% of medical litigation cases in high-income countries involve imaging, of which nearly 80% relate to trauma [2]. The morbidity and mortality associated with missed fractures are well documented [7], [8], [9], [10].

The accuracy of trauma radiographs reported by junior doctors has been extensively researched in the last half century [4,[11], [12], [13], [14], [15], [16], [17], [18], [19], [20], [21], [22], [23]]. Previous studies have largely been performed in well-resourced environments and have included all levels of healthcare facilities. A variety of research methods have been utilized, yielding a wide range (32% - 99%) of reporting accuracy for junior colleagues.

Despite most injuries worldwide occurring in low- and middle-income countries [24], there has been very limited work on the accuracy of trauma radiograph reporting in such settings. The two studies to date have assessed fracture detection by junior South African (SA) doctors working in tertiary hospitals and have recorded accuracies of 68% [25] and 89% [26], respectively. However, there has been no study in a district hospital setting in Africa.

Such a study is important, since a review of radiological resources in Southern, East, and West Africa reveals a broad network of district hospitals with plain radiographic equipment [27], [28], [29], [30]. Furthermore, the District Health System (DHS) [25] has been adopted as the vehicle for delivery of comprehensive primary health care in many African countries.

The aim of this study was an audit of the accuracy of after-hour acute trauma-radiograph reporting by doctors in the emergency centre (EC) of a district hospital in Africa.

## Methods

This retrospective descriptive study was conducted at a large SA district hospital, with more than 300 beds and a busy EC, treating more than 3000 patients monthly.

The EC is staffed by physicians with a wide range of experience, including interns, post-internship “community service” doctors, medical officers, registrars (trainee specialists), and qualified specialists. Each patient's initial management is based on the individual assessment of a junior EC doctor, who requests and interprets first-line investigations, including radiographs. Imaging requests are at the sole discretion of the examining physician and are informed by clinical findings and the mechanism of trauma. There is subsequent formal specialist-review of patient management.

The hospital has a fully digital imaging platform, with a filmless and paperless radiology department, but no after-hour radiologist reporting service. An electronic timestamp is generated for each investigation. All studies are available for clinical viewing at any hospital workstation immediately after completion. All images are stored on the institutional picture archiving and communication system (PACS), which thus represents a comprehensive imaging database. In addition, the full medical record for each patient is available digitally on the electronic medical record.

The study was conducted over two consecutive pre-COVID weekends in February 2020. For study purposes, a weekend extended from 16h00 Friday - 08h00 Monday; day shift was from 08h00 - 20h00, and night shift from 20h00 - 08h00 the following day.

The admission radiographs of trauma patients aged 18 years or older, who were managed in the EC over the study weekends, were included. All follow-up and non-trauma radiographs, those performed on down-referred patients, and any examination on patients less than 18 years of age, were excluded.

Any injury caused by a gunshot or stab with a knife, broken bottle, or other sharp object was deemed penetrating. An injury was classified as blunt if due to a punch, kick, or blow with any blunt object such as a rock, plank, or concrete slab. Traffic-related injuries, characterised by their high-velocity and propensity for polytrauma, included motor vehicle accidents (MVA) and pedestrian vehicle accidents (PVA). Community assault, a distinct mechanism of injury specified in the referral details, involves polytrauma induced by whipping and direct blows with multiple different blunt objects. It infrequently involves penetrating injuries.

There was independent reporting of all study radiographs by two radiologists blinded to clinical details: a radiology consultant with 11 years’ experience and a senior radiology registrar with 5 years’ experience.

Images with technical limitations such as under-penetration, over-penetration, or inappropriate positioning were excluded.

Radiographs were assessed for fractures, dislocations, joint effusions, pneumomediastinum, pneumothorax, haemothorax, pulmonary opacification, widened mediastinum and pneumoperitoneum. All abnormal findings were subjectively stratified as “easily identified” or “subtle”. Results were captured on a customized, forced-choice reporting template. Discrepancies between radiologists were resolved by consensus.

Details of patients’ age, gender, mechanism of injury (MOI), time of examination, and injuries detected by EC doctors were subsequently included on the spreadsheet.

The axial skeleton included the skull, facial bones, and the spine; the appendicular skeleton comprised the upper/lower limbs and pelvis. Chest and abdominal radiographs were assessed separately.

The study endpoint was the overall accuracy of initial EC doctor radiographic interpretation, with the radiologist report considered the “gold-standard”. For each radiograph, the final radiologist report was compared with the initial EC interpretation. Injuries were deemed detected if documented in the EC clerking notes or included in clinical details on subsequent imaging requests. Conversely, an undetected injury was neither specified in the initial EC notes nor on any additional imaging request.

The overall accuracy, sensitivity, specificity, positive predictive value (PPV) and negative predictive value (NPV) for initial EC radiographic interpretation were calculated with 95% confidence intervals. The Fischer's Exact Test assessed the association between reporting accuracy and anatomical region, time of investigation, mechanism of injury and the number of abnormalities per radiograph. Undetected injuries were classified as “clinically significant” or “not clinically significant”, the former if non-detection impacted clinical management and potentially influenced patient outcome.

This study was approved by the Health Research Ethics Committee of Stellenbosch University and the National Health Research Database.

## Results

Study population: Seventy-nine (*n* = 79) patients were included (m = 59; 75%), with median age 31 years (interquartile range: 27-37 years). Injuries were *penetrating* (stabs, panga wounds and gunshots) in almost half the patients (*n* = 39, 49%), due to *blunt* trauma in approximately a quarter (*n* = 19, 24%), vehicle-related in almost one-fifth (*n* = 14, 18%), and due to community assault in the remainder (*n* = 7, 9%).

Inter-observer Agreement: Initial interpretation yielded 97% (*n* = 136/140) inter-observer agreement (*n* = 136/140). The four minor discrepancies on the initial read were resolved by consensus.

Analysis by radiograph (Table 1)**:** One hundred and fifty-three (*n* = 153) admission trauma-related radiographs were acquired in the study period. Thirteen (*n* = 13, 8.5%) were excluded on technical grounds. One hundred and forty (*n* = 140) radiographs were thus included.

**Table 1:** Analysis by radiograph.

	n(%)	Normal(n)	Abnormal(n, %)	Correct Diagnosis(n, %)	False Negative(n)	FalsePositive(n)	True Negative(n)	True Positive(n)	Sensitivity (%)	Specificity(%)	Accuracy (%)	PPV(%)	NPV (%)	p-value (n, %)
**Type of Investigation**														
Appendicular skeleton	70 (50)	44/70	26/70 (37)	54/70 (77)	15	1	43	11	42 (23–63)	98 (88-100)	77 (66-86)	92 (62–100)	74 (67-80)	0.02
CXR	52 (37)	35/52	17/52 (33)	41/52 (79)	10	1	35	6	38 (15–65)	97 (85-100)	79 (65-89)	86 (44–98)	78 (70-84)	
Axial skeleton	14 (10)	10/14	4/14 (29)	11/14 (79)	2	1	10	1	33 (1–91)	91 (59–100)	79 (42-95)	50 (8–92)	83 (69-92)	
AXR	4 (3)	2/4	2/4 (50)	2/4 (50)	2	0	2	0	0 (0–84)	100 (16–100)	50 (7-93)	n/a	50 (50-50)	
Total	140 (100)	91/140	49 (35)	108/140(77)	29	3	90	18	38 (25–54)	97 (91–99)	77 (69-84)	86 (65–95)	76 (71-80)	
**Time of investigation**														
Day shift	65 (46)	39/65	26/65 (40)	45/65 (69)	18	2	39	6	25 (10–47)	95 (83–99)	69 (57–80)	75 (39–93)	68 (63–73)	0.04
Night shift	75 (54)	52/75	23/75 (31)	63/75 (84)	11	1	51	12	52 (31–73)	98 (87–100)	84 (69–90)	92 (62–99)	78 (70–84)	
**Mechanism of Injury**														
Penetrating Injury	51 (36)	30	21/51 (41)	37/51 (73)	12	2	30	7	37 (16–62)	94 (79–99)	73 (58–84)	78 (45–94)	71 (64–78)	< 0.01
Traffic-related Injury	38 (27)	27	11/27 (29)	30/38 (79)	8	0	27	3	27 (6–61)	100 (87–100)	79 (63–90)	100 (37–100)	77 (70–83)	
Blunt Injury	37 (26)	24	13/37 (35)	32/37 (86)	5	0	24	8	62 (32–86)	100 (86–100)	86 (71–95)	100 (69–100)	83 (71–91)	
Community Assault	14 (10)	10	4/14 (29)	9/14 (64)	4	1	9	0	0	90 (56–100)	64 (35–87)	0 (0–95)	69 (65–73)	

Images of the appendicular skeleton (*n* = 70, 50%) and chest (*n* = 52, 37%) together accounted for almost ninety percent (*n* = 122, 87%) of the workload, while studies of the axial skeleton (*n* = 14, 10%) and abdomen (*n* = 4, 3%) constituted the remaining. Just over half the investigations (*n* = 78, 56%) were performed during the evening shift.

Almost two-thirds of the radiographs (*n* = 91, 65%) had no abnormality. Of the 49 abnormal studies (*n* = 49/140, 35%), just over half (*n* = 27/49, 55%) had a single abnormality, more than a third (*n* = 19/49, 39%) two abnormal findings, and less than ten percent (*n* = 3/49, 6%) three abnormalities.

One-hundred and eight radiographs (*n* = 108) were correctly interpreted, representing overall accuracy of 77% (95% CI), with 38% sensitivity (95% CI = 25 – 54%) and 97% specificity (95% CI = 91 – 99%).

Normal radiographs were interpreted with almost complete accuracy (*n* = 90/91, 99%), compared to 37% (*n* = 18/49) for abnormal studies.

Reporting sensitivity and specificity tended to be associated with the site of injury. Performance was best for abnormalities of the appendicular skeleton, decreasing sequentially for the chest, axial skeleton and abdomen (p = 0.02).

Similarly, performance tended to be associated with the mechanism of injury, with blunt trauma achieving the highest, and community assault the lowest sensitivity and specificity, respectively (p < 0.01).

Night shift reporting tended to be more accurate than day shift (p = 0.04). Accuracy was also associated with the number of abnormalities on a single film. Images demonstrating one, two and three-or-more abnormalities were interpreted correctly in 48%, 26% and 0% of cases, respectively (p < 0.01).

Analysis by abnormality (Table 2):

**Table 2:** Analysis by abnormality.

Injury No.	Gender (M/F)	Age (Years)	Time of Investigation	X-ray	Mechanism of Injury	Findings / Diagnosis	Detected (Y/N)	Subtle (Y/N)	Clinically Significant (Y/N)
1	F	20	21:14	L Elbow	Penetrating Injury	Ulna fracture	N	N	Y
2	M	40	11:03	Pelvis	Blunt Injury	Right femur fracture	Y	N	Y
3	M	40	11:03	R Hip	Blunt Injury	Right femur fracture	Y	N	Y
4	M	31	14:50	CXR	Traffic-related Injury	Left rib fracture	Y	N	Y
5						Left pneumothorax	Y	N	Y
6	M	31	14:50	Pelvis	Traffic-related Injury	Left femur fracture	Y	N	Y
7	M	31	14:50	AXR	Traffic-related Injury	Left rib fracture	N	N	N
8						Left femur fracture	N	N	Y
9	M	31	13:31	CXR	Penetrating Injury	Right haemothorax	Y	Y	Y
10	M	39	10:20	CXR	Penetrating Injury	Right haemothorax	N	Y	Y
11	M	31	15:58	CXR	CA	Left haemothorax	N	Y	Y
12	F	57	6:31	L Tibia / Fibula	Blunt Injury	Left tibia fracture	Y	N	Y
13						Left fibula fracture	Y	N	Y
14						Left ankle fracture	N	N	Y
15	F	57	6:31	L Ankle	Blunt Injury	Left tibia fracture	Y	N	Y
16						Left fibula fracture	Y	N	Y
17	M	29	1:22	CXR	Penetrating Injury	Right pneumothorax	Y	N	Y
18						Right haemothorax	Y	N	Y
19	M	31	2:11	CXR	Penetrating Injury	Left pneumothorax	Y	N	Y
20						Left haemothorax	Y	N	Y
21	M	28	20:07	CXR	Penetrating Injury	Left pneumothorax	Y	N	Y
22						Left haemothorax	Y	N	Y
23	M	25	12:11	CXR	Penetrating Injury	Left rib fracture	N	Y	N
24						Left pneumothorax	Y	N	Y
25						Left haemothorax	Y	N	Y
26	M	64	9:04	R Ankle	Assault NOS	Right tibia fracture	N	N	Y
27						Right fibula fracture	Y	N	Y
28	M	22	10:09	CXR	Penetrating Injury	Left haemothorax	N	Y	Y
29	F	26	16:46	CXR	Penetrating Injury	Right pulmonary laceration	N	N	N
30	F	40	1:48	R Wrist	Traffic-related Injury	Right radius fracture	Y	N	Y
31						Right ulna fracture	N	Y	Y
32	M	33	3:45	L Knee	Traffic-related Injury	Left knee effusion	N	Y	Y
33						Left tibia fracture	Y	N	Y
34	F	27	10:19	R Ankle	Blunt Injury	Left fibula fracture	Y	N	Y
35	M	29	22:34	CXR	Penetrating Injury	Left pulmonary laceration	N	N	N
36	M	31	19:08	AXR	Penetrating Injury	Air under the diaphragm	N	Y	Y
37	M	31	11:06	Facial bones	Penetrating Injury	Left mandible fracture	Y	Y	Y
38	M	31	11:06	R Wrist	Penetrating Injury	Right carpal dislocation	N	N	Y
39						Right ulna fracture	Y	N	Y
40	M	31	8:51	CXR	Penetrating Injury	Right haemothorax	Y	N	Y
41	M	48	21:55	Pelvis	Blunt Injury	Right femur fracture	Y	N	Y
42	M	48	21:55	R Femur	Blunt Injury	Right femur fracture	Y	N	Y
43	M	39	10:37	Facial bones	Assault NOS	Right mandible fracture	Y	N	Y
44						Left mandible fracture	N	Y	Y
45	F	31	11:42	CXR	Penetrating Injury	Left pneumothorax	Y	N	Y
46						Left haemothorax	N	N	N
47	M	26	17:46	R Hand	Penetrating Injury	Phalanx fracture	Y	N	Y
48						Phalanx dislocation	N	N	Y
49	M	27	4:33	Pelvis	Traffic-related Injury	Right pelvic fracture	Y	N	Y
50	M	27	4:33	R Hip	Traffic-related Injury	Right ilium fracture	Y	N	Y
51						Right ischium fracture	N	N	Y
52	M	27	19:37	L Foot	Penetrating Injury	Left metatarsal fracture	N	Y	Y
53	M	23	16:02	Facial bones	Penetrating Injury	Left mandible fracture	Y	N	Y
54	M	23	13:58	R Femur	Traffic-related Injury	Right femur fracture	Y	N	Y
55						Right tibia fracture	N	N	Y
56	M	21	12:46	C-spine	CA	C-spine dislocation	N	Y	Y
57	M	21	12:46	CXR	CA	Right scapula fracture	N	Y	Y
58	F	36	22:40	L Radius / Ulna	Assault NOS	Left elbow dislocation	Y	N	Y
59	F	36	4:39	CXR	Traffic-related Injury	Widened mediastinum	N	Y	Y
60	F	24	7:01	L Radius / Ulna	Penetrating Injury	Left radial fracture	Y	N	Y
61	M	46	12:14	R Radius / Ulna	Blunt Injury	Left Radius fracture	Y	N	Y
62						Left Ulna fracture	N	N	Y
63						Left Radio-ulna dislocation	N	N	Y
64	M	33	3:37	CXR	Penetrating Injury	Right pneumothorax	Y	N	Y
65	M	35	10:03	Pelvis	CA	Right pelvic fracture	N	Y	Y
66	M	38	23:29	Pelvis	Traffic-related Injury	Right pelvic fracture	N	N	Y
67						Right hip dislocation	Y	N	Y
68	M	38	23:29	R Hip	Traffic-related Injury	Right hip dislocation	Y	N	Y
69						Right acetabular fracture	N	N	Y
70	M	28	5:53	CXR	Penetrating Injury	Left pneumothorax	Y	N	Y
71						Left haemothorax	N	N	N
72	M	20	0:11	R Shoulder	Assault NOS	Right clavicular fracture	Y	N	Y
73	M	46	0:29	R Radius / Ulna	Blunt Injury	Right radius fracture	Y	N	Y
74						Right ulna fracture	N	N	Y

Overview: The 49 abnormal radiographs demonstrated seventy-four (*n* = 74) individual pathological findings. More than two-thirds of abnormalities (*n* = 51, 69%) involved the bony skeleton, including fractures (*n* = 43/74, 58%), dislocations (*n* = 7/74, 9%) and a solitary knee effusion (*n* = 1/74, 1%).

More than eighty percent of fractures (*n* = 36/43, 84%) involved the appendicular skeleton (long bones = 25/43, 58%; pelvis = 6/43, 14%; shoulder 2/43, 5%; hand = 1/43, 2%; foot = 1/43, 2%; ankle = 1/43, 2%), while axial skeleton (face = 4/43, 9%) and chest fractures (ribs = 3/43, 7%) each accounted for less than ten percent of such abnormalities.

Similarly, more than eighty percent of dislocations (6/7, 86%), involved the appendicular skeleton. There was a solitary cervical spine (axial skeleton) dislocation.

Soft tissue injuries to the chest (*n* = 22, 30%), accounted for almost a third of injuries, including haemothorax (*n* = 11, 15%), pneumothorax (*n* = 8, 11%), pulmonary opacification (*n* = 2, 3%) and mediastinal widening (*n* = 1, 1%).

There was a single case of pneumoperitoneum.

Ease of identification: Eighty percent of abnormalities (*n* = 59/74, 80%) were classified as “easily identifiable”. Almost three-quarters (*n* = 42/59, 71%) involved the bony skeleton, with the remainder (*n* = 17/59, 29%) thoracic soft tissue injuries.

Most “easily identifiable” skeletal abnormalities (*n* = 36/42, 86%) were fractures; a small minority (*n* = 6/42, 14%) were dislocations.

Pneumothoraces (*n* = 8) and haemothoraces (*n* = 7) accounted for more than eighty percent of easily identifiable thoracic soft-tissue injuries (17/17, 88%).

Detection by abnormality: 43 of 74 abnormalities (*n* = 43/74, 58%) were correctly identified, including 29/51 (57%) skeletal injuries and 14/22 (64%) thoracic soft tissue abnormalities. The pneumoperitoneum was not detected.

All hand fractures (*n* = 1/1, 100%), three quarters of facial fractures (*n* = 3/4, 75%), more than 70% of long bone fractures (*n* = 18/25, 72%), half the shoulder girdle fractures (*n* = 1/2, 50%), less than half the dislocations (*n* = 3/7, 43%), and one-third of the pelvic (2/6, 33%) and rib (1/3, 33%) injuries were correctly identified on initial assessment. The foot and ankle fractures together with the knee effusion were not detected.

All pneumothoraces (*n* = 8) and more than half the haemothoraces were detected (*n* = 6/11, 55%). Pulmonary opacification (*n* = 0/2) and mediastinal widening (*n* = 0/1) were not identified.

**Fig. 1:**
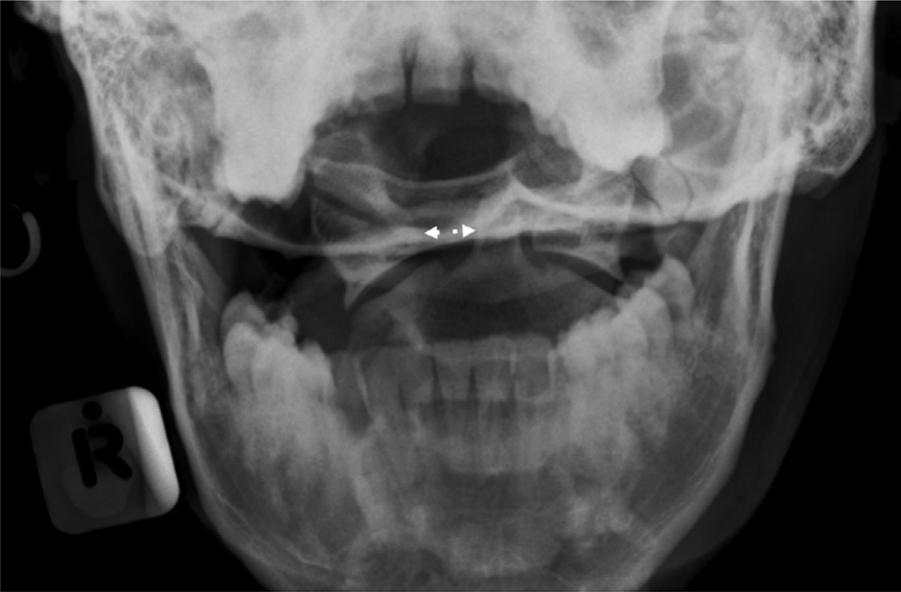
False-negative. *Note:* The widened right transverse ADI was not detected (white double-headed arrow). This was considered a ‘subtle, clinically significant’ error since it indicates atlanto-axial instability and warrants further cross-sectional imaging.

Detection by ease of identification: Forty-one (*n* = 41/59, 69%) “easily identifiable” injuries were detected, of which 28/41 (68%) involved the bony skeleton and 13/41 (32%) the chest soft tissue. Conversely, 15 radiographic abnormalities (*n* = 15/79, 20%) were considered “subtle” (15/79), of which just 2/15 (13%) were identified (Figure 1).

False positives: There were six false positive findings: three fractures (right mandibular body, humeral shaft and metacarpal), two pneumothoraces and one haemothorax.

The missed fractures were not clinically significant since management was not impacted. In the case of the false positive right mandibular fracture, there was a true-positive contralateral mandibular fracture that required definitive CT imaging, which demonstrated a normal right mandible. The missed humeral fracture occurred in conjunction with a true-positive ulna fracture, which was appropriately managed with a back-slab and follow-up imaging. The metacarpal fracture was also managed conservatively.

One false positive pneumothorax was not clinically significant, as the patient had an ipsilateral haemothorax for which an intercostal drain was placed.

One false positive pneumothorax and the haemothorax were clinically significant as they were managed with intercostal drainage.

**Fig. 2:**
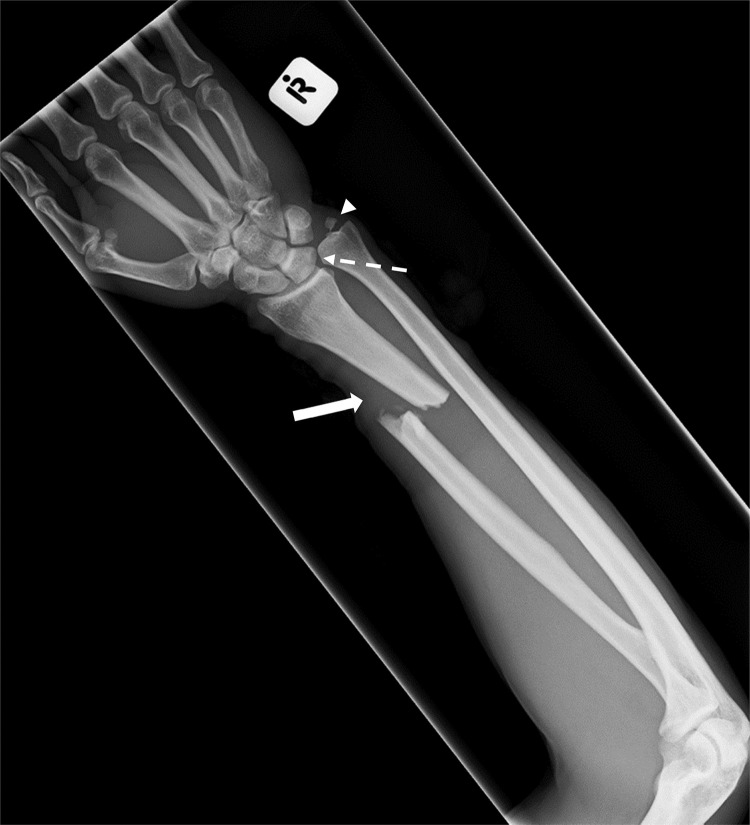
False negative. *Note:* Galeazzi fracture-dislocation of the right forearm, with fracture of the distal third of the radius and disruption of the distal radioulnar joint. The radial fracture was detected (solid white arrow), but the radio-ulnar dislocation (dashed white arrow) and ulnar styloid fracture (white arrow head) were missed. Deemed ‘easily-detectable, clinically significant”.

**Fig. 3:**
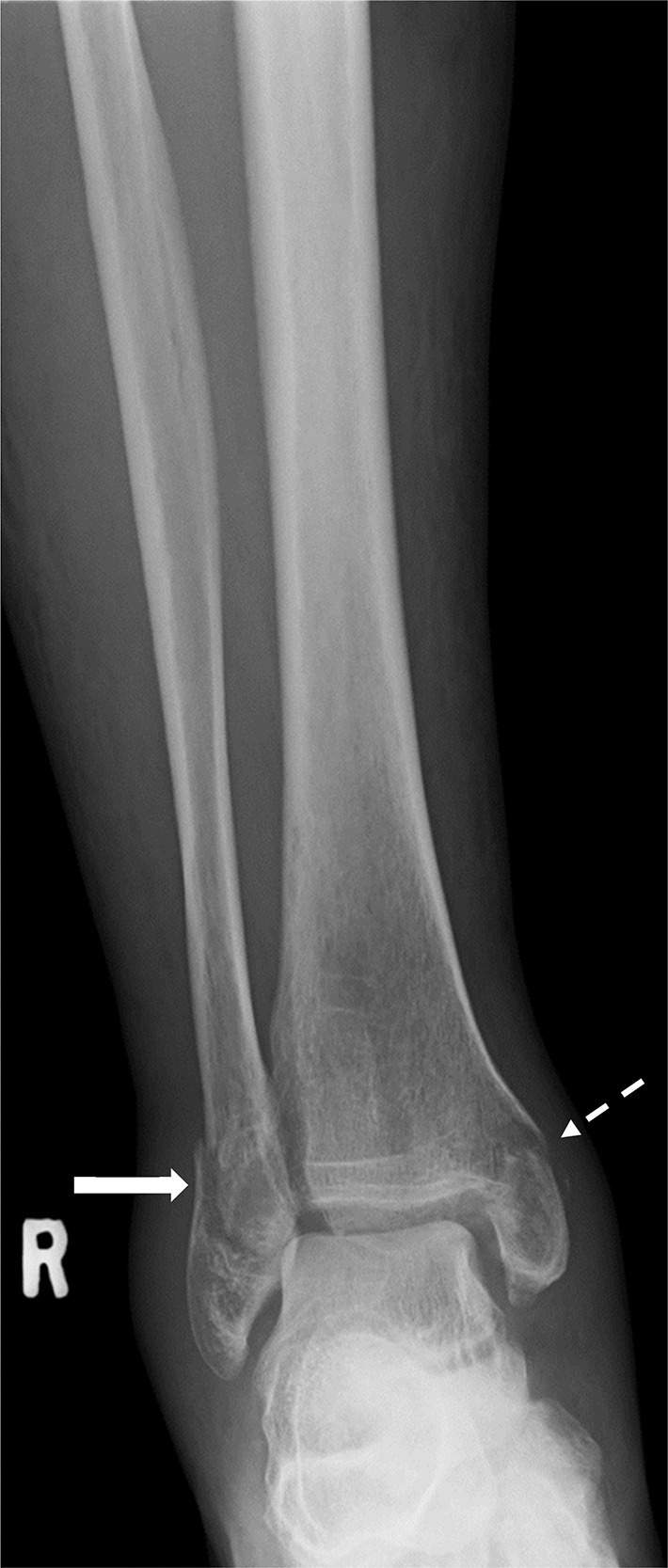
False negative. *Note:* Weber B, Lauge Hansen 4 ankle fracture. Only the fibula fracture was detected (solid white arrow). The medial malleolar avulsion was missed (dashed white arrow). Deemed ‘easily-detectable, clinically significant’.

Analysis by clinical significance: Almost three-quarters (*n* = 27/37, 73%) of reporting errors were clinically significant, including 25/31 (81%) undetected abnormalities and 2/6 (33%) spurious findings (Figures 1, 2, 3).

The 6 undetected abnormalities deemed insignificant included 2 haemothoraces with correctly identified pneumothoraces for which intercostal drains were appropriately sited, 2 unidentified pulmonary lacerations, and 2 undetected rib fractures required only conservative management.

One-third of spurious findings (*n* = 2/6, 33%) were clinically significant, involving a single patient and resulting in unnecessary placement of an intercostal drain.

## Discussion

To the best of our knowledge, this work represents the most detailed analysis of acute trauma radiograph reporting by junior doctors in any setting. It thus makes an important contribution to the broad body of existing literature on this subject. It is also the first study in the setting of the African district hospital, providing important new insights into an under-researched area of traumatology and emergency medicine. It documents key baseline data in this context.

Firstly, our cohort had a higher proportion of abnormal radiographs (35%) than reported (8% - 25%) [14,17,18,31] in other settings. Williams [22] and Vincent [18] have stressed the importance of this parameter when assessing reporting accuracy, noting its omission in most published work. They caution that studies involving a high proportion of normal radiographs may be biased towards improved outcomes if false negatives were simply recorded as a percentage of the total examinations. Our finding that normal radiographs were reported with almost complete accuracy (90/91, 99%) supports this contention. Furthermore, it has been suggested [31] that high proportions of normal trauma radiographs reflect inappropriate imaging requests and warrant more stringent referral criteria. Although the optimum ratio of normal to abnormal trauma radiographs is moot, the percentage of abnormal radiographs in our study arguably reflects conservative utilization of imaging in a resource constrained environment.

Secondly, almost half (22/49, 45%) the abnormal radiographs showed more than one pathological finding, a detail not routinely included in the body of literature. Our finding that reporting accuracy decreases with the number of abnormalities on a film (p < 0.01) is intuitive but highlights its importance. The challenge of making a correct and complete diagnosis in the multiply injured has been previously documented [32,33]. However, the so-called “satisfaction of search” [34], whereby pathology on the same radiograph remains undetected after identification of an initial abnormality, is a well-recognised radiological pitfall that has not been highlighted in the EC literature to date.

Thirdly, approximately one-in-five (15/79, 20%) radiographic abnormalities were considered “subtle”, and thus required substantial radiological training and experience for identification. The importance of this detail, which is mostly not reported, has been stressed by Tachakra [16] and borne out by our finding that only 13% of “subtle” abnormalities were identified, compared to 69% of those deemed “easily identifiable”.

Fourthly, almost three-quarters of reporting errors (27/37, 73%) were considered clinically significant, which is substantially higher than the 6% - 44% reported in previous studies [14,17,18,22,26]. This may reflect the complexity of trauma in our environment. South Africa is recognised as one of the most violent countries in the world, with an exceptionally high prevalence of interpersonal violence, as evidenced by almost sixty percent of our cohort being victims of penetrating injury or community assault [35].

The finding that reporting accuracy is associated with anatomical region (p=0.02), type of trauma (p=<0.01) and time of day (p=0.04) is important. It suggests caution should be exercised when comparing performance across studies while highlighting key variables that may impact outcomes. Our study nonetheless provides useful baseline data in the African context.

We have shown that injuries to the appendicular skeleton and chest are most common in our setting, together accounting for almost 90% of all trauma radiographs. We have also shown that reporting sensitivity is higher for these anatomical regions than for those less commonly injured (axial skeleton and abdomen). It could be that the sheer volume of appendicular skeleton and chest trauma hones reporting skills.

Additionally, we have shown that radiographs resulting from the more conventional blunt trauma tend to be reported with greater sensitivity than those from more complex mechanisms of injury, such as penetrating trauma, motor vehicle accidents and community assault. In their classic manuscript, Tachakra and Becket [16] defined four scenarios accounting for radiological errors by junior doctors in the accident and emergency unit. The first was insufficient care when reviewing the radiograph, with missed fractures at the site of maximum tenderness being a prime example. The second was unexpected radiological findings, such as fractures distant from the site of maximum tenderness. The third was radiological inexperience, whereby abnormalities were either identified, but considered normal variants, or not identified due to their subtlety. The fourth scenario was radiographic interpretation without clinical examination. The risks inherent at the time of staff changeover were highlighted. The scenario of staff at the start of a shift reviewing the radiographs of patients for whom they had assumed responsibility, but had not examined personally, was noted as particularly high risk. It is unclear to what extent this scenario contributed to the disparity in reporting accuracy between the day and night shift in our study.

The finding that night shift was more accurate than day shift reporting is certainly counter-intuitive. The explanation is not immediately apparent. The determinants of sensitivity in trauma reporting remain poorly understood and will require evaluation in future work. However, review of Table 1 will show that there was a higher proportion of normal studies performed in the night shift (52/75, 69%) than the day shift (39/65, 60%). This may have contributed to this finding.

Notwithstanding the limitations of comparing studies of radiological reporting performance, the 77% overall accuracy achieved by EC staff in this study is well within the 44% - 99% range [ref] documented in the international literature for non-radiologist reporting of conventional trauma radiographs in the clinical environment [13,22,36].

A major strength of this study was its foundation on two robust digital databases, one being an electronic medical record and the other a picture archiving and communication system. This allowed comprehensive retrieval of imaging and clinical data. In addition, the study was designed to facilitate the most detailed analysis to date of overall accuracy of initial EC doctor radiographic interpretation. As such it reported parameters not included in previous manuscripts, such as sensitivity, specificity, PPV, NPV. It also included parameters inconsistently documented in the literature, such as the proportion of radiographs with any abnormality, more than one abnormality, and subtle abnormality. It also assessed the association between reporting accuracy and anatomical region, mechanism of injury and the time of day. Future studies in this domain could utilize this methodology.

The study was limited by retrospective data acquisition and a relatively small sample size. Additionally, it did not assess final patient management or outcomes. It is thus possible that radiological errors by junior doctors were corrected during subsequent consultant ward rounds or consultations.

This study has identified key factors that impact the accuracy of trauma radiograph reporting in our setting. It also underscores the challenge of such work and highlights the need for further training of junior doctors in this area of clinical practice. Future work in this domain should focus on assessing the impact of such training on the reporting accuracy.

## Dissemination of Results

The results will be presented to the local health facilities and the local health authorities as well as the education committee involved in the training of medical students. The findings of this research will be presented at national and international emergency medicine conferences.

## Author Contributions

Authors contributed as follow to the conception or design of the work; the acquisition, analysis, or interpretation of data for the work; and drafting the work or revising it critically for important intellectual content: YML contributed 50%; RDP 25%; SO 15%; FCH and SL contributed 5% each. All authors approved the version to be published and agreed to be accountable for all aspects of the work.

## Declaration of Competing Interest

The authors declare no conflicts of interest.
